# Numerical analysis of pile group, piled raft, and footing using finite element software PLAXIS 2D and GEO5

**DOI:** 10.1038/s41598-023-42783-x

**Published:** 2023-09-23

**Authors:** Firanboni Fituma Chimdesa, Firaol Fituma Chimdesa, Nagessa Zerihun Jilo, Anand Hulagabali, Olusola Emmanuel Babalola, Tiyasha Tiyasha, Krishnaraj Ramaswamy, Adarsh Kumar, Suraj Kumar Bhagat

**Affiliations:** 1 Department of Civil Engineering, School of Civil and Environmental Engineering, Ambo University, Ambo, Ethiopia; 2 Department of Civil Engineering, College of Architecture and Civil Engineering, Addis Ababa Science and Technology University, Addis Ababa, Ethiopia; 3 The National Institute of Engineering (NIE), Mysuru, 570008 India; 4 Faculty of Civil Engineering, Ton Duc Thang University, Ho Chi Minh City, Vietnam; 5 Department of Civil Engineering, School of Engineering and Computing, Dev Bhoomi Uttarakhand University, Dehradun, India; 6 Department of Mechanical Engineering, College of Engineering and Technology, Dambi Dollo University, Dembi Dolo, Ethiopia; 7 Institute of Natural Sciences and Mathematics, Ural Federal University, Ekaterinburg, 620002 Russia

**Keywords:** Solid Earth sciences, Engineering

## Abstract

Foundation plays a vital role in weight transfer from the superstructure to substructure. However, foundation characteristics such as pile group, piled raft, and footing remain unfolded due to their highly non-linear behaviour in different soil types. Bibliography analysis using VOSvierwer algorithm supported the significance of the research. Hence, this study investigates the load-bearing capacity of different types of foundations, including footings, pile groups, and piled rafts, by analyzing experimental data using finite element tools such as PLAXIS 2D and GEO5. The analysis involves examining the impact of various factors such as the influence of surcharge and the effect of different soil types on the load-bearing capabilities of the different types of foundation. For footing, parametric investigations using PLAXIS 2D are conducted to explore deformational changes. Pile groups are analyzed using GEO5 to assess their factor of safety (FOS.) and settling under various criteria, such as pile length and soil type. The study also provides insight into selecting the right type of foundation for civil engineering practice. Findings showed that different soil types have varying deformational behaviours under high loads with sandy soil having less horizontal deformation than clayey soil. Also, it was observed that increasing the pile thickness by 50% resulted in a reduction of 13.88% in settlement and an improvement of 16.66% in the FOS. In conclusion, this study highlights the importance of professionalism, exceptional talent, and outstanding decision-making when assessing the load-bearing capabilities of various foundation types for building structures.

## Introduction

Foundation is a substructure Component that serves as a weight transfer mechanism for the superstructure's supporting columns and walls. Footing being the foundation's backbone, also delivers important benefits such as avoiding excessive or unequal settlement, rotation, and providing enough resistance to sliding and overturning. Large-scale structures can now be built because of new developments in civil engineering. These structures can apply substantial loads to the soil. Pile groups are employed to enhance the depth of footings and transfer loads to soil layers with higher bearing capacity (such as thick gravel, gravelly sand, hard clay, or rock) in cases where the soil's rigidity and bearing capacity are inadequate^1^. When dealing with substantial loads imposed by large-scale structures, piled raft foundations (PRFs) offer a viable solution when shallow footings are insufficient^2,3^. PRFs improve the ultimate load-bearing capacity of shallow footings, reduce settlements, and minimize bending moments within the raft. This system combines the load-bearing capacities of the soil, raft, and piles into a composite structure. The behaviour of PRF in a multilayered soil profile is significantly influenced by the thickness of the flexible raft^4,5^. To ensure the safe design of such footings, it is crucial to comprehend the intricate soil-structure interaction behaviours that influence the behaviour of PRFs^1,2^. The optimal pile arrangement can be chosen to minimize differential settlement by taking into account loading intensity and serviceability limit factors^6^.To ensure the long-term functionality of organic soils in construction projects, various soil modification techniques are employed, including methods like cutting and replacing, displacement, or pre-compression^7^. Pile foundations are also employed to address issues about the insufficient strength and excessive settling of organic soil^8^. When shallow footings cannot accommodate these loads, PRFs are typically used as a substitute^1^. In the past few years, combined piled raft foundation technique has gained popularity as an enhanced foundation option, particularly for high-rise structures^9–13^. A raft, a sequence of piles, and subsoil comprise a hybrid piled raft foundation^14^. Piled raft foundations have gained significant popularity in recent years due to their ability to combine the bearing capacities of piles and rafts. Extensive research has been undertaken to ensure the reliability and cost-effectiveness of designing such foundations through rigorous analytical, numerical, and experimental studies^8,15^. Various foundations are considered depending on the type of construction. Shallow foundations are chosen over deep foundations if the soil carrying capacity is adequate for the weight of the structure^16,17^. When the soil carrying capacity is insufficient to support the load of the structure, a deep foundation or a mix of the two is used. Footing is a substructure component that serves as a weight transfer mechanism for the superstructure's supporting columns and walls. Since footing is the foundation's backbone, it also delivers important benefits such as avoiding excessive or unequal settlement, and rotation and providing enough resistance to sliding and overturning. Pile foundations are one sort of deep foundation that has the benefit of resisting uplift pressures in the same way as they take compression forces in the other direction via skin friction^18,19^. When the soil's carrying capacity is limited and large settlements are expected, piles are a preferable solution. A pile cap is built during foundation pile design to allow for the connection of pile heads and convenient load transmission. A pile cap is intended for structural section capacity, with the cap playing a significant role if it comes into direct contact with the foundation soil. A pile group is formed when many pile ends are joined together by a common structural member known as a pile cap.

High-rise structures, bridges, power plants, and oil tankers might all be built on top of piled rafts.^20^. PRF is a three-part load-bearing structure composed of piles, rafts, and subsoil which is more strong and safe. The raft and pile share most of the superstructure weight, decreasing settling. The raft has an appropriate rolling limit and reduces differential settling, although it settles excessively. As a result, the pile-side raft is used as a piled raft to set a good bearing limit and reduce settling within significant, fair, and safe cut-off points. Davis and Poulos were the first to propose a stacked-raft foundation. For piled raft foundations, somewhat stiff or medium clay soil strata, and thick sand soil strata are excellent^21^. Soft clays at the surface, as well as soft compressible layers at relatively short depths, are unsuitable for stacked raft foundations. Several previous theoretical and experimental studies have been carried out to better understand the performance of mixed piled raft foundations by varying variables such as soil properties, raft stiffness, pile number, pile spacing, and applied load level^22–25^.

An investigation was conducted to analyze the behaviour of strip footings on the surface of uniform clay in an undrained state, employing elastoplastic finite element analysis^26^. The study revealed that the ultimate bearing capacity of strip footings decreases as the consistency of clay transitions from hard to soft and with an increase in inclined load. To further explore this topic, a numerical analysis was performed utilizing PLAXIS 2D^27^. The study determined the bearing capacity of strip footings on a sand slope and estimated the bearing capacity of rectangular footings situated on the top surface of the model ground using PLAXIS 2D software. Moreover, a group of researchers proposed a new finite element methodology to examine the behaviour of flexible combined pile–raft foundation (CPRF) positioned in layered soil, in a displacement-based framework^28^. The research reveals that the thickness of flexible raft plays an important role to understanding the behaviour of flexible CPRF in layered soil.

PLAXIS is a finite element software designed for geotechnical engineering analysis. It offers advanced capabilities for modelling complex soil-structure interaction problems, including pile rafts and footings. It also offers specialized geotechnical features such as modelling soil behaviour, accounting for pile-soil-raft interaction, and simulating the load response of foundations. On the other hand, the key advantage of GEO5 is that the user may experiment with more options and analytical approaches to determine the most likely pile foundation behaviour and then compute the total bearing capacity or settlement of a single pile or a pile group. The use of GEO5 for pile analysis and PLAXIS 2D for pile-raft allows for robust and detailed Finite Element Analysis modelling of the complex soil and structural behaviour, providing a more accurate representation of real-world conditions.

**Figure 1:**
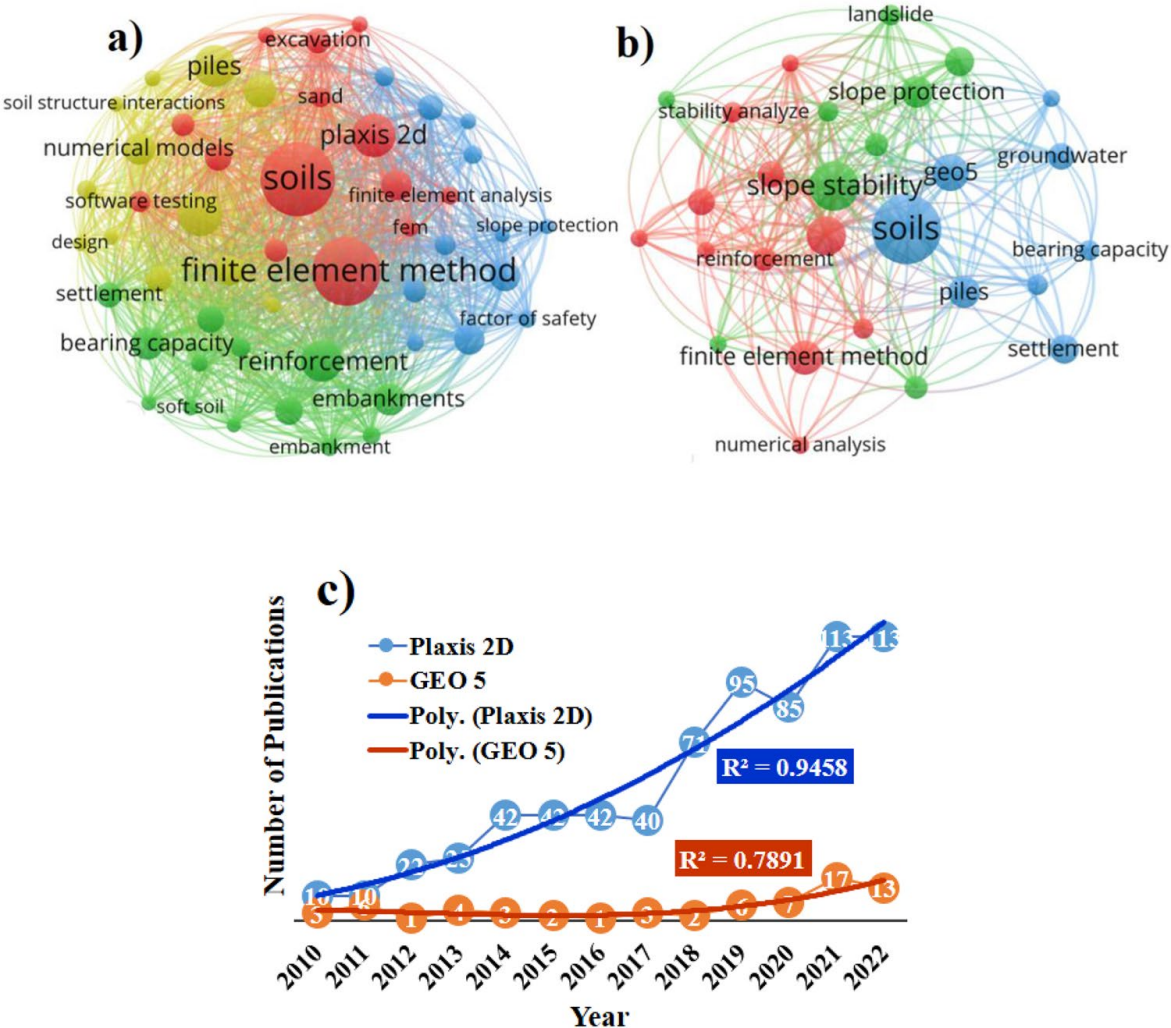
HYPERLINK "sps:id::fig1||locator::gr1||mediaobject::0"Total keywords used in the Scopus index database literature based on application of (**a**) PLAXIS 2D and (**b**) GEO5 software to analyze the finite element for Pile Group, Piled Raft, and Footing; and their (**c**) progress over time span of the conducted literature.

It is worth mentioning that the VOSviewer algorithm was applied using 889 published research articles from the Scopus indexed database, Fig. 1a was generated to present the major keyword co-occurrence i.e., 51 items reached the threshold (22 occurrence) value among total 4844 keywords, presenting the significant established PLAXIS 2D software application for finite element analysis. Figure 1b was generated to present the major keyword co-occurrence i.e., 37 items reached the threshold (5 occurrence) value among total 1105 keywords, presenting the significant established GEO5 software application for finite element analysis. Moreover, the progress of the adopted research over the past decade has been demonstrated and it clearly presented the importance of using different finite element analysis tools such as PLAXIS 2D, and GEO5 (Fig. 1c). However, the GEO5 needs more attention over the upcoming times to set the comprehend conclusion.

This research aims to conduct a comprehensive numerical analysis of pile groups and foundation systems using advanced software tools. Specifically, the study explores the effects of various parameters, including pile thickness, pile shape, soil type, ground water table, and pile length, on the behaviour, and performance of pile groups using GEO5 software. Additionally, the research will analyze the response of piled raft and footing foundations under different loads, soil types, and surcharge loads, utilizing PLAXIS 2D FEM-based software to assess factors such as the factor of safety (FOS), settlement, and deformation. By conducting this rigorous analysis, the study intends to provide valuable insights into the design and behaviour of foundation systems, helping to inform more effective and reliable engineering practices in the field of geotechnical engineering in the design of foundation elements of structures.

The manuscript is divided into four sections. The introduction is followed by the material and methodology applied in this study. Detailed information is provided about the data, model, and numerical analysis of the pile group and footing using finite element software like GEO5 and PLAXIS 2D. The next section contains the result and discussion about the outcome from the GEO5 software considering the FOS, length of the pile, thickness of the pile cap, depth of the groundwater table, shape of the pile cap, and different soil types. This section also dealt with the results from PLAXIS 2D software, with detailed information about various deformations in pile raft properties. The third subsection of "Result and discussions" gives information about the performance of PLAXIS 2D software used for studying footing properties, with detailed information about various deformations in footing properties.

## Materials and methods

### Data selection

The study area of this research is Ambo town, situated in the West Shewa Zone of the Oromia Region, to the west of Addis Ababa, Ethiopia. Its geographical coordinates are approximately 8°59′ N latitude and 37°51′ E longitude. When it comes to modelling an earth system, it is crucial to have reliable data to prevent any modelling errors. To ensure accuracy, this study conducted geotechnical tests to gather field data for modelling and simulation purposes.

To assess the qualities of various soil layers in the study area, a comprehensive geotechnical investigation program was conducted, reaching a depth of up to 40 m. The research involved collecting soil samples from the study region and performing a range of field tests, such as the standard penetration test. Additionally, several laboratory studies were conducted, including analyses of grain size distribution, Atterberg limits, permeability, and unconfined compression test as per the ASTM standard^29,30^. To evaluate and determine the soil characteristics of the study area, the obtained soil research data was compared with previous study findings and standard codes of ASTM^31^. For a broad understanding of those standards, their references can be explored^30,32,33^. The analysis identified three main soil types in the research region: sandy soil, gravel soil, and fine-grained soil. Relevant information regarding the unit weight, Poisson's ratio, stiffness modulus, shear strength, and starting stress coefficient for each of these soil types are provided in Table 1 (sandy soil), Table 2 (gravel), and Table 3 (fine-grained soil).^34^ and^35^ can be consulted for further details. Based on the consistency and soil type, recommendations were applied to estimate two crucial elastic parameters: the elastic modulus and Poisson's ratio^36^ provide specific details on this methodology. Subsequently, these parameters were utilized to calculate the shear strength parameters.

**Table 1:** Sandy soil parameters used as modelling input.

Soil parameters	Units	SW	SP	SM	SC
Poisson’s ratio, v	–	0.28	0.28	0.3	0.35
Unit weight, $\upgamma$	kN/m^3^	20	18.5	18	18
Deformation modulus, E_def_	MPa	30–60	15–35	5–15	4–12
Effective shear parameters					
The angle of internal friction, Φ′	Degrees	34–39	32–35	28–30	26–28
Cohesion of soil, C′	kPa	0	0	0–10	4–12
Design bearing capacity					
Foundation width < 0.5 m (Rd)	kPa	195	160	175	125
Foundation width < 1.0 m (Rd)	kPa	325	225	225	175
Foundation width < 3.0 m (Rd)	kPa	520	390	300	225
Coefficient of structural strength	m	0.3	0.3	0.3	0.3
Below ground water table	m	0.2	0.2	–	–

*SW* well-graded sand, *SP* poorly-graded sand, *SM* silty sand, *SC* clayey sand.

**Table 2:** Gravel parameters used as the modelling input.

Soil parameters	Units	GW	GP	GM	GC
Poisson’s ratio, v	–	0.2	0.2	0.3	0.3
Unit weight, $\upgamma$	kN/m^3^	21	20	19	19.5
Deformation modulus, E_def_	MPa	250–390	100–190	60–80	40–60
Effective shear parameters					
The angle of internal friction, Φ′	Degrees	36–41	33–38	30–35	28–32
Cohesion of soil, C′	kPa	0	0	0–8	2–10
Design bearing capacity					
Foundation width < 0.5 m (Rd)	kPa	325	360	250	150
Foundation width < 1.0 m (Rd)	kPa	520	420	300	200
Foundation width < 3.0 m (Rd)	kPa	650	550	400	250
Coefficient of structural strength	m	0.3	0.3	0.3	0.3
Below ground water table	m	0.2	0.2	–	–

*GW* well-graded gravel, *GP* poorly-graded gravel, *GM* silty gravel, *GC* clayey gravel.

**Table 3:** Fine-grained parameters used as the modelling input.

Soil parameters	Units	CH	CL	MH	ML
Poisson’s ratio, v	–	0.42	0.4	0.4	0.4
Unit weight, $\upgamma$	kN/m^3^	20.5	21.0	21	20
Deformation modulus, E_def_	MPa	2–4	3–6	3–5	3–5
Effective shear parameters					
The angle of internal friction, Φ′	Degrees	13–17	17–21	15–19	19–23
Cohesion of soil, C′	kPa	2–8	8–16	4–10	8–16
Total shear parameters					
The angle of internal friction, Φ	Degrees	0	0	0	0
Cohesion of soil, C	kPa	40	50	50	60
Foundation width < 3 m (Rd)	kPa	80	100	100	150
Coefficient of structural strength	m	0.2	0.2	0.2	0.2
For E_def_ < 4Mpa; not over consolidated	m	0.1	0.1	0.1	0.1

*CH* high compressible clay, *CL* low compressible clay, *MH* high compressible silt, *ML* low compressible silt.

### Finite element simulation

Two-dimensional finite element simulations were used to analyze the settlement, deformation, and FOS of a pile group, piled raft, and footings. The numerical simulation was conducted using the finite element method and the packaged software GEO5 and PLAXIS 2D. To replicate the material used in both applications, the Mohr–Coulomb (MC) model was employed. As the mesh size in finite element analysis drops, so does the convergence to real-world findings. When the mesh is very tiny, the analysis takes longer. The analysis cannot be finished owing to convergence error, even though the solution time lowers as the mesh size rises. After experimenting with various mesh sizes, the ideal mesh size was discovered. We utilized medium mesh in this situation. Pile length, ground-water table, soil type, pile cap thickness, and other variables all influence pile group performance^37–42^. Various approaches can be used to study the influence of these factors. Numerical analysis is the process of numerically examining the influence of those factors on the performance of piled raft and footing using PLAXIS 2D FEM-based software, although this would be challenging due to the complicated link between working circumstances and soil pile interaction that must be considered.

#### Constitutive model

In this paper, the focus lies on incorporating the Mohr–Coulomb (MC) model, which is a nonlinear model extensively employed to estimate soil behaviour. The MC model is well-known and favoured due to its simplicity and ability to provide accurate results. It takes into account five input variables: elasticity modulus (E), Poisson's ratio (v), cohesion (c), internal friction angle, and dilatancy angle (ψ). These variables collectively contribute to the overall assessment of soil characteristics^43^.

The following equation can be used to define MC model

1 $$\tau =c-\sigma tan\varphi$$

where $\tau$ presents the shear stress, $\sigma$ presents the normal stress, c is the cohesion of the soil material and $\varphi$ is the angle of internal friction.

#### Boundary condition

The modelled boundary conditions are designed based on certain assumptions. In this case, the vertical boundaries are assumed to have freedom of movement in the vertical direction but are constrained horizontally. On the other hand, the bottom horizontal boundary is considered to be completely fixed.

#### Mesh generation

**Figure 2:**
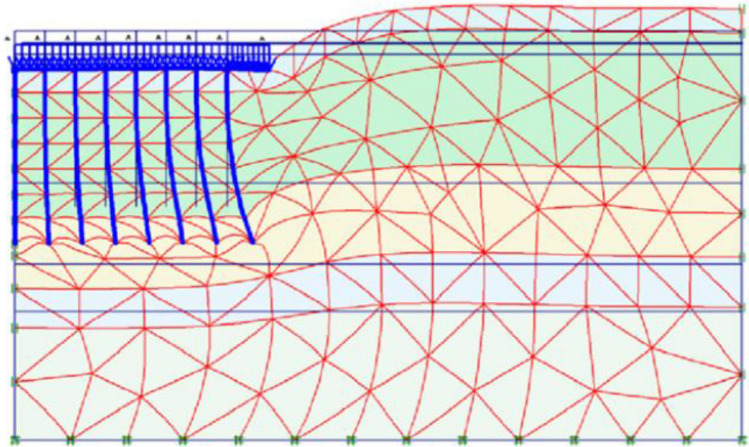
Finite element mesh for piled raft.

**Figure 3:**
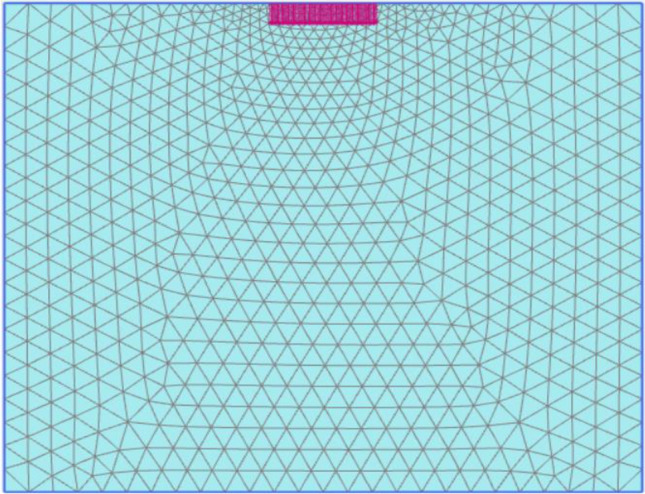
Finite element mesh for footing.

The mesh is generated automatically, and specific enhancements have been implemented to achieve smaller mesh sizes in areas where stress and deformation are concentrated, such as piled raft, footing, and pile group locations^44,45^. Finite element calculations require dividing the geometry into elements. In this study, the components illustrated in Figs. 2 and 3 were constructed using a mesh comprised of 15-node triangular elements within PLAXIS 2D. After creating the model geometry and assigning material properties to all clusters and structural objects, the geometry inputs are divided for finite element calculations. To mitigate the impact of mesh dependency in finite element modelling scenarios involving variations in the number, length, and placement of geogrid layers, a modified mesh was utilized.

### Numerical analysis of pile group using finite element software GEO5

**Figure 4:**
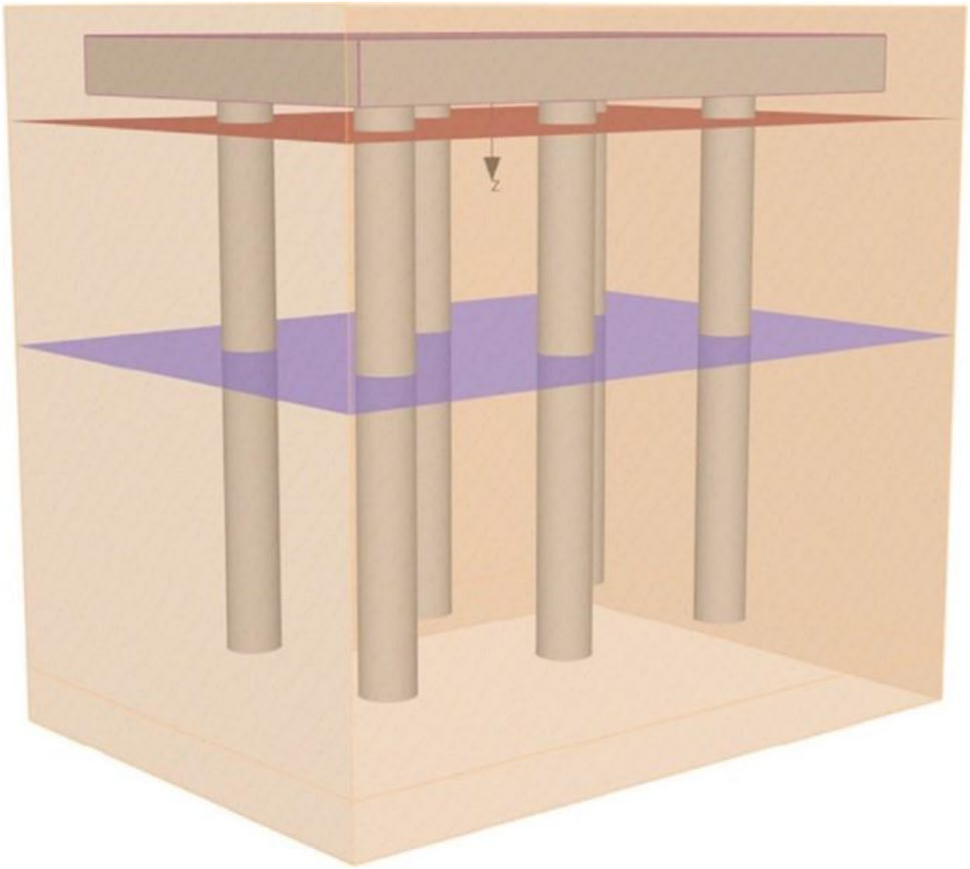
3D arrangement of piles in a group in GEO5.

**Figure 5:**
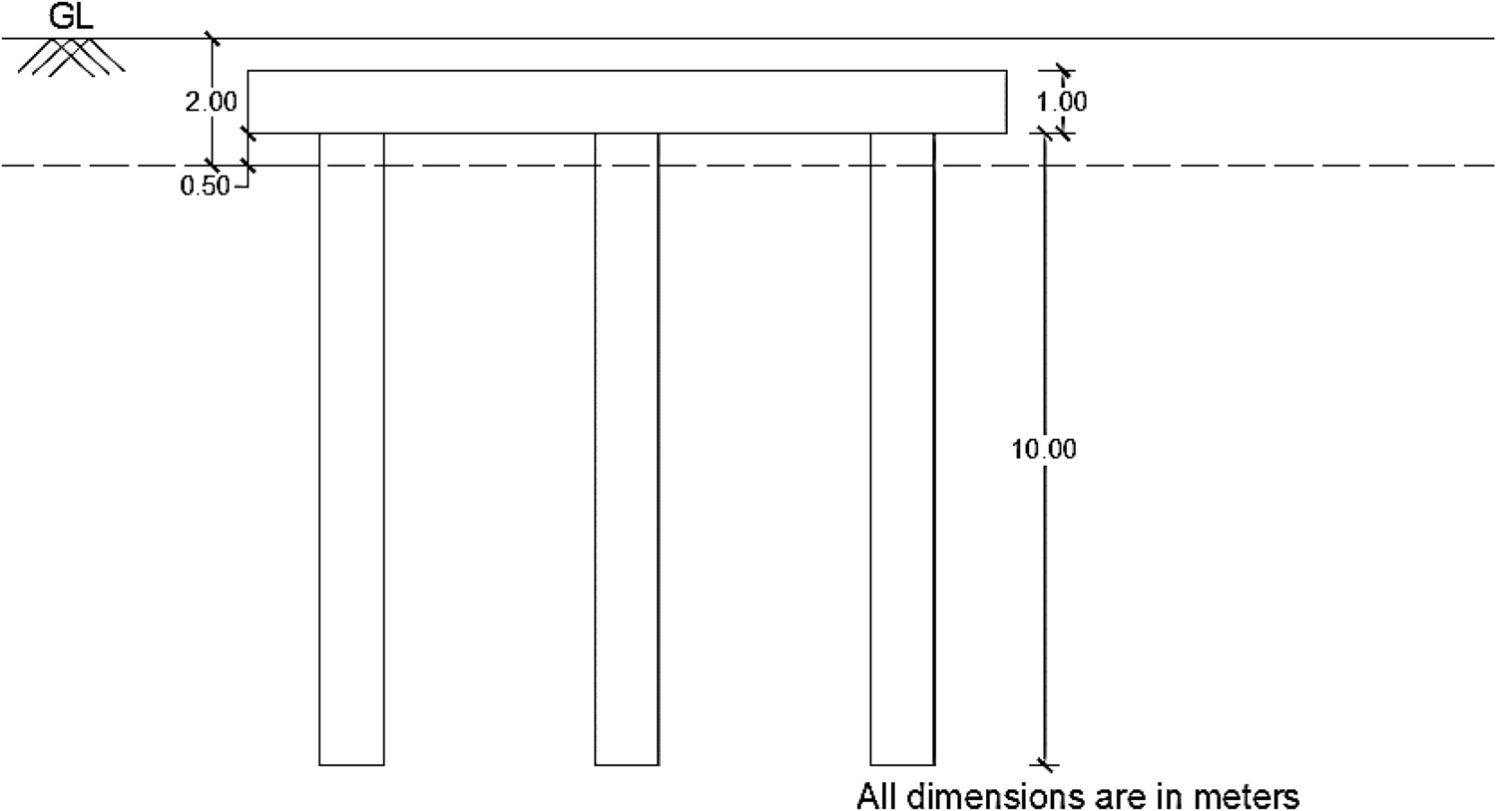
Cross-sectional view of the pile group in GEO5.

**Figure 6:**
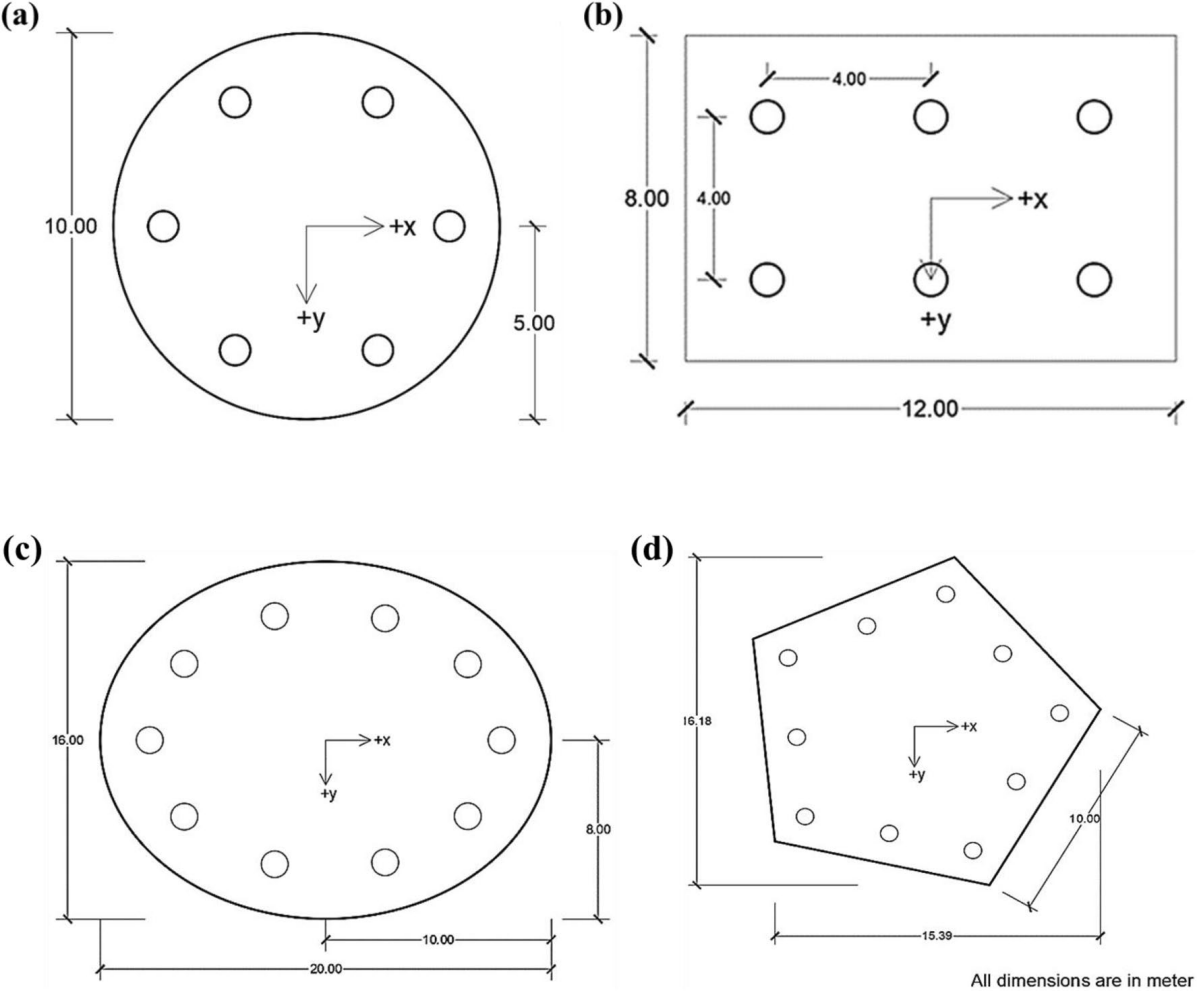
Different shapes of pile cap used for analysis in GEO5 (**a**) Circular pile cap, (**b**) rectangular pile cap, (**c**) spherical pile cap, (**d**) hexagonal pile cap.

In this model, a 3D model of a pile group and soil was created using the numerical modelling platform of GEO5^46^, as depicted in Fig. 4 In the current experiment, the software provided semi-infinite boundary conditions at the top of the model, while vertical sides have roller support, and the bottom has all degrees of freedom constrained. A pile group consisted of six heaps with lengths of 10 m and depths of 1 m. Figure 5 displays a 0.5 m pile head offset and a depth of 2 m from the ground surface. Figure 6 depicts a schematic diagram of the pile group's strategy. The pile cap is 12 × 8.0 m^2^. Each pile is 1 m in diameter and 10 m in depth. The x-axis has three piles, whereas the y-axis has two rows. In the x and y directions, the spacing between piles is 4 m. Table 4 shows the RCC of piles and cap specifications.

**Table 4:** Input loads for analysis.

No	N (kN)	M_x_ (kN m)	M_y_ (kN m)	H_x_ (kN)	H_y_ (kN)
1	2500	150	200	100	75
2	1755	92	114	57	43
3	2170	110	165	85	60
4	1523	77	116	59	42
5	1850	105	120	65	30
6	1295	74	86	32	13
7	1920	135	160	95	70
8	1637	96	108	64	23

The soil types and general features are indicated in Tables 1, 2, 3 and 4. These parameters have been applied as input parameters for modelling. All other factors are constant while the targeted parameter is investigated to identify its effect. A Pile Group is a collection of piles that strengthen the soil while also carrying the applied weight down to deeper, stronger soil layers. Friction or end bearings can cause load resistance.

The load capacity efficiency factor (ξpr) can be assessed for any operational load and the associated settlement level by utilizing Eq. (2). Equation (3) represents the resultant predictive equation for FOS_pr_^47^.

2 $$FOS_{{{\text{pr}}}} = \, \left[ {{2}.{25}\left( {s_{{{\text{avg}}}} /D_{{\text{p}}} } \right) \, + \, 0.0{2}\left( {s/D_{{\text{p}}} } \right) \, - \, 0.00{12}\left( {L_{{\text{p}}} /D_{{\text{p}}} } \right) \, + \, 0.{525}} \right]\left( {Q_{{{\text{ur}}}} /Q_{{{\text{ult}}}} + Q_{{{\text{pg}}}} /Q_{{{\text{ult}}}} } \right)$$

3 $$FOS_{{{\text{pr}}}} = \xi_{{{\text{pr}}}} \left( {FOS_{{{\text{ur}}}} + FOS_{{{\text{pg}}}} } \right)$$

4 $$FOS_{{{\text{ur}}}} = Q_{{{\text{ur}}}} - {\text{ ult}}/Q$$

5 $$FOS{\text{pg }} = Q{\text{pg}} - {\text{ult}}/Q$$

The mobilized safety factor for the PR (FOSpr) can be determined using Eq. (3)^48^. This equation considers the separate mobilized safety factors for UR (FOSur) and PG (FOSpg), which correspond to the desired average settlement value of up to 0.35% Br. To calculate FOSur and FOSpg for any working load (Q), Eqs. (4) and (5) are utilized. It is important to note that Qpg-ult is estimated based on individual pile failure. Additionally, the ξ_pr_ can be evaluated for any working load and the corresponding settlement level. The resulting predictive equation for FOS_pr_ is expressed in Eq. (2).

The final pile cap shapes and their model dimensions for the numerical analysis are rectangular, circular, spherical, or hexagonal, as shown in Fig. 6 (all measurements are in meters (m)).

### Numerical analysis of piled raft with finite element software PLAXIS 2D

**Figure 7:**
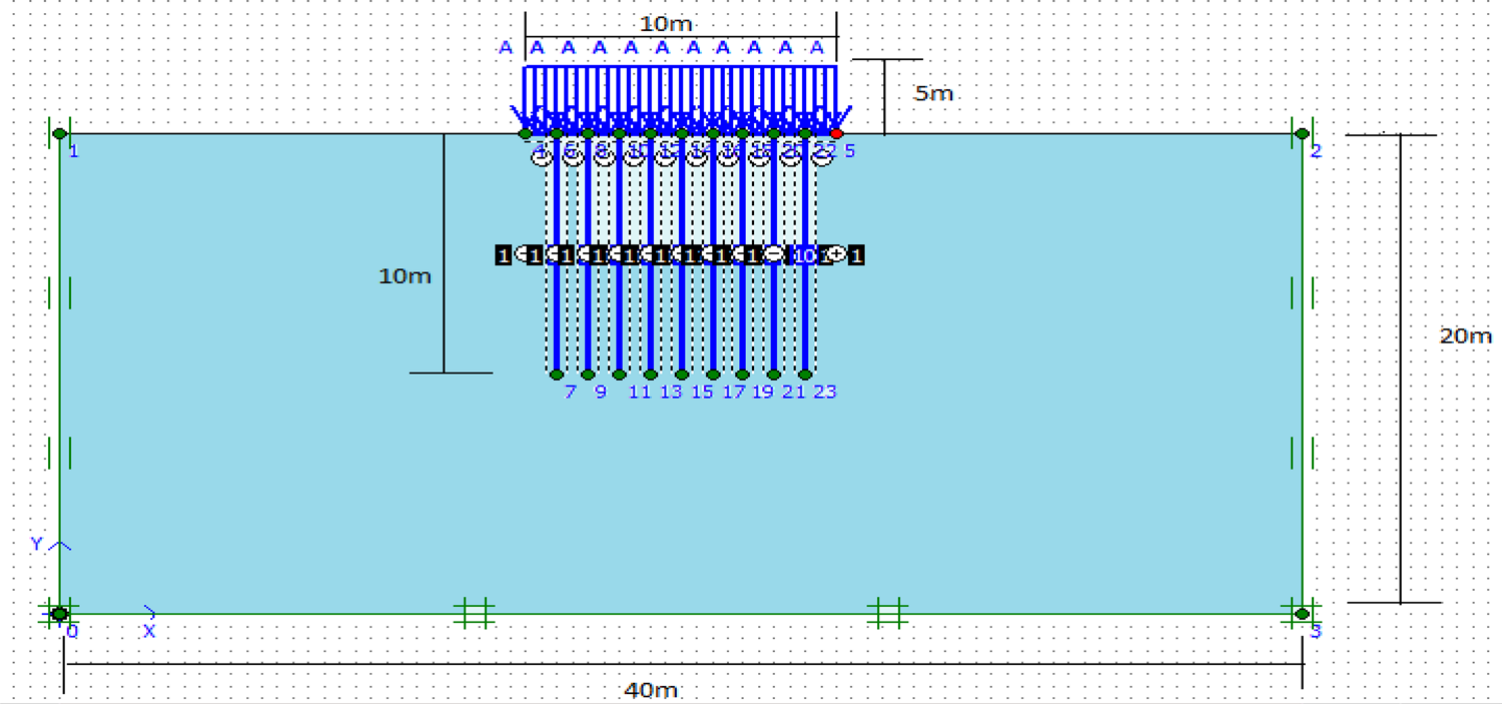
Model of piled raft in PLAXIS 2D.

The finite element program PLAXIS 2D is critical software for doing geotechnical analyses quickly, conveniently, and precisely^49^. Even though a comprehensive three-dimensional analysis is recommended for accuracy, a two-dimensional finite element analysis is employed in this study. This is because the two-dimensional analysis tends to overestimate system settlement by up to 30% and pile load by up to 10%. Despite these limitations, it offers the benefits of time savings and simplicity. Notably, the studies conducted by^25^, and Chore et al. ^26^ demonstrate similar approaches. The primary objective of this research is to validate the findings from laboratory model tests and evaluate parameters that cannot be explored through the laboratory model. The analysis of axially loaded piled rafts is typically considered a three-dimensional problem due to the complex loading and geometry involved. However, symmetrical techniques can be employed to simplify the analysis and reduce it to two dimensions. To present a piled raft foundation in a simplified manner, PLAXIS 2D version 8.2 utilizes a planar strain model^5^. In the context of foundation analysis, a three-dimensional piled raft can be simplified and represented as a two-dimensional strip piled raft. Various models, such as linear elastic and nonlinear plane strain finite element models, can be utilized to study the behaviour of a piled raft. One commonly employed approach involves using a six-noded triangular element to simulate the piled raft and the surrounding soil. Furthermore, for analytical purposes, the pile rows are often divided into strips, which facilitates a more efficient and manageable assessment of the piled raft system. In the simulation, the piled raft and the mud were represented using a six-noded triangular element, while specific strips were created to represent the pile rows. In order to account for the dimensions of the pile and raft, it is necessary to simplify the in-plane row of piles into a plane strain pile. This simplification involves considering an equivalent pile modulus of deformation based on the number of piles in the row. Figure 7 illustrates the numerical model used to address the relevant issues and incorporates the various elements required to simulate real-world scenarios. The finite element model's geometry was carefully designed to match the dimensions of the experimental model. The displacements in the x- and y-axes at the bottom, as well as the x-axis at the sides, are fixed at zero. To facilitate a streamlined mesh optimization, the cluster surrounding the piled raft was generated using the ''refine cluster" option during mesh generation. The model consists of three distinct parts: soil clusters represented by 6-node elements, rafts and piles represented by plate/beam elements, and an interface element to capture the interaction between the soil and structural elements. Tables 1, 2, 3 and 4 provide a comprehensive list of the material properties utilized in the finite element analysis conducted for this problem statement. Also, Table 5 provides the RCC properties of Pile and Cap. Entry of input data is required in the software analysis, followed by the setting of the model architecture which includes general setting and defining standard constant parameters such as geometry, loads (distributed loads in our case), materials (soil types), and meshing, as well as additional parameters that influence the behaviour of piled raft (studied parameters).

**Table 5:** The RCC properties of pile and cap^46^.

Parameters	Units	Values
Compressive strength, f_ck_	MPa	20
Tensile strength, f_cr_	MPa	2.20
Elasticity modulus, E_c_	MPa	30,000
Shear modulus, G	MPa	12,500
Longitudinal steel tensile strength, f_yk_	MPa	500
Transverse steel tensile strength, f_yk_	MPa	500

### Numerical analysis of footing using finite element software PLAXIS 2D

The numerical modelling in this research employed the specialized geotechnical analysis software called PLAXIS 2D. This software was utilized to assess the bearing capacity and failure analysis of deep rectangular footings positioned at the center of the soil model. The primary focus of the study was to evaluate the ultimate bearing capacity of a square footing, both at the surface and at a depth of 5 m within the same soil layer. PLAXIS software provides two types of elements: a six-noded triangular element and a fifteen-noded triangular element, which enable more precise computations of strains and stresses. To simulate realistic field conditions, appropriate boundary conditions were applied to the model. The vertical movement allowed for the left and right boundaries while maintaining zero displacements in the horizontal direction. To mitigate boundary effects resulting from load application, a substantial distance was maintained between the foundation and the vertical and horizontal boundaries of the model. The geometry of the finite element soil model used in the analysis had dimensions of 10B × 10B, where 'B' denotes the width of the foundation. The behaviour of the soil beneath the footing was represented using the Mohr–Coulomb (MC) model, which is an elastic–plastic constitutive model.

**Figure 8:**
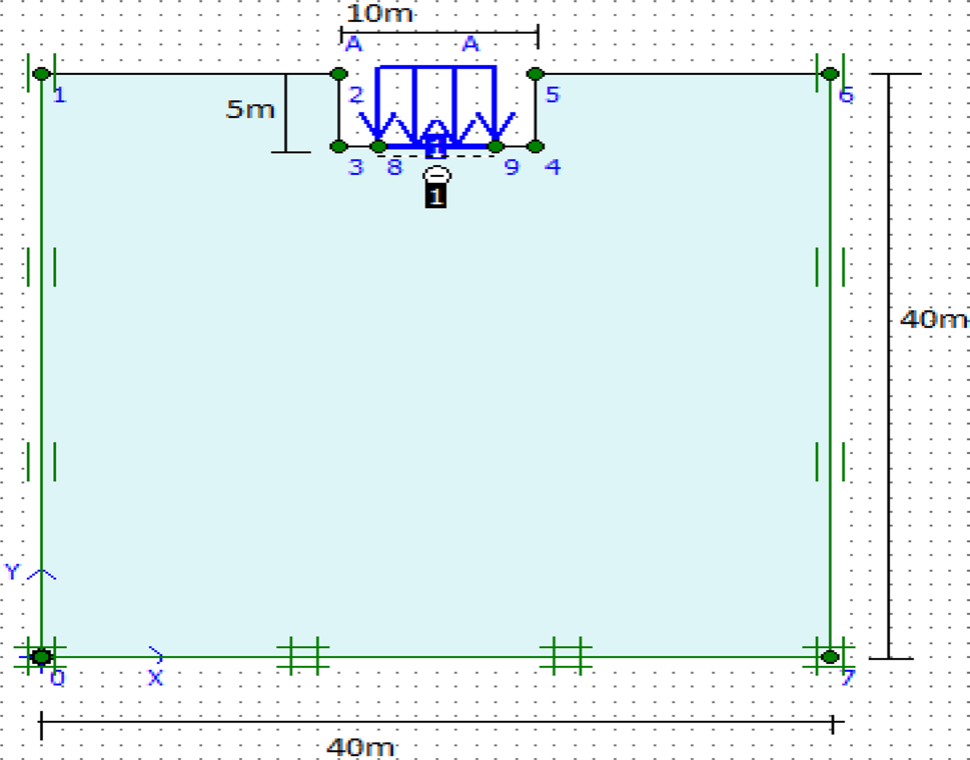
Model of footing using FEM-based tool PLAXIS 2D.

A rectangular foundation with a width of 10 m is treated as a soil foundation contact area similar to that of a square foundation. The foundation analysis employed a mesh composed of 15-node triangular elements, which is commonly utilized for such investigations. The footing was modelled with the flexibility to accurately represent its behaviour. The characteristics of the soil and footing utilized in the analysis can be found in Table 1 through Table 4. The analysis aimed to examine the deformation of the footing under varying surcharge (pressure) and the impact of different soil types on deformation. Figure 8 displays the model of the footing created using the finite element method tool, PLAXIS 2D.

## Result and discussions

### Result of study of pile group using GEO5 software

#### Variation of a factor of safety and settlement concerning the length of the pile

In the present study, we focused on investigating the impact of pile length variation on a pile group consisting of 3 × 2 piles embedded in silty gravel soil. The loads applied to the pile length of 5 m, 10 m, 15 m, 20 m, and 25 m were recorded and analyzed. The test results revealed an interesting trend. As the length of the piles increased, the factor of safety also increased. This indicates that longer piles offer a higher level of stability and support. Additionally, the settlement of the pile group decreased as the length of the piles increased. This suggests that longer piles experience less settlement, which is desirable in terms of structural integrity.

**Figure 9:**
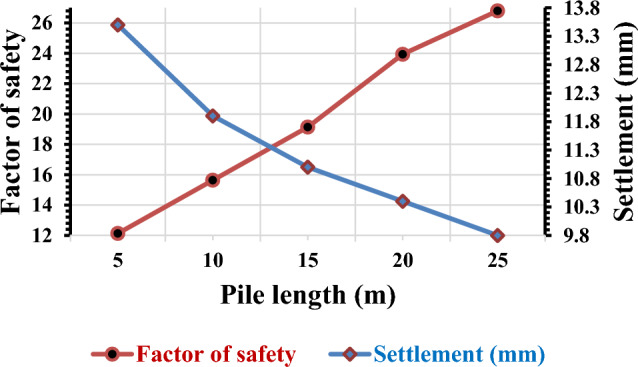
Pile group settlement and factor of safety variation due to change in length of the pile.

For a visual representation of the relationship between the change in pile length and the settlement of the pile group, as well as the FOS, please refer to Fig. 9. This figure illustrates the impact of varying pile lengths on the settlement and factor of safety of the pile group. Overall, the findings of this study emphasize the importance of considering pile length when designing pile groups in silty gravel soil, as longer piles can significantly enhance stability and reduce settlement. In fact, increasing the original pile length by 50% results in a 23.07% decrease in settlement and a 25% increase in the factor of safety. These numbers demonstrate the extent to which settlement is influenced by the pile length.

#### Variation of a factor of safety and settlement concerning the thickness of pile cap

**Figure 10:**
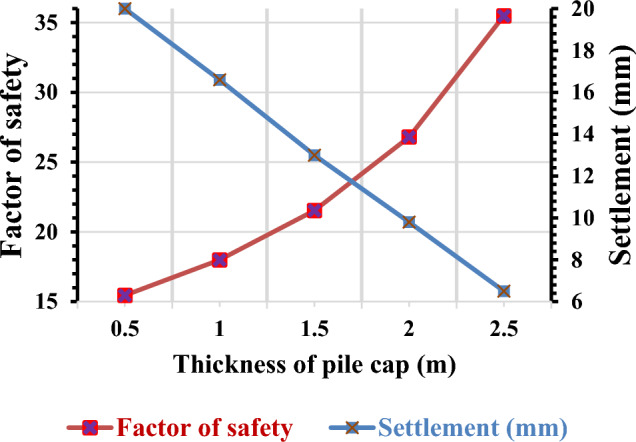
Pile group settlement and factor of safety variation due to change in thickness of pile cap.

In a further study, the impact of altering the thickness of the pile cap on structural stability and settlement was studied. The pile cap plays a crucial role in connecting a group of piles and preventing the structure from settling. To analyze this, pile cap thicknesses of 0.5 m, 1 m, 1.5 m, 2 m, and 2.5 m were considered. The findings, shown in Fig. 10, revealed an interesting correlation. As the thickness of the pile cap increased, the factor of safety also increased. This implies that thicker pile caps provide greater structural stability and resistance against settling. Additionally, the settlement of the structures was observed to decrease with the increase in pile cap thickness.

These results suggest that modifying the thickness of the pile cap can have a significant impact on the structural integrity and settlement behaviour of the foundation system. The study highlights the importance of considering appropriate pile cap thicknesses to ensure the long-term stability and performance of structures. Notably, increasing the pile thickness by 50% resulted in a reduction of 13.88% in settlement and an improvement of 16.66% in the factor of safety.

#### Variation of a factor of safety and settlement concerning the depth of groundwater table

**Figure 11:**
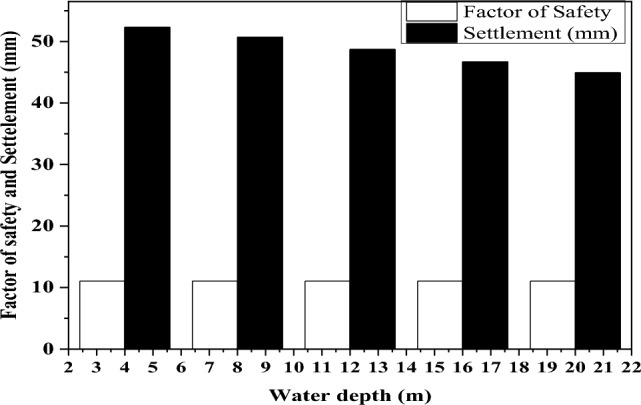
Pile group settlement and FOS variation due to change in the groundwater table.

The rise in the groundwater table significantly impacts the performance of the pile foundation system. As the soil water content increases, the soil tends to become weaker, which limits the load-carrying capacity of the soil and can potentially lead settlement of the structure^6^. To ensure the construction of a safe and cost-effective structural design, it is crucial to evaluate the depth of the water table. Figure 11 illustrates the effects of changes in the groundwater table on pile group settlement and FOS. During the study, groundwater depths were measured at intervals of 4 m, 8 m, 12 m, 16 m, and 20 m from the ground surface.

The numerical results indicate that the FOS remains relatively consistent across different water depths. However, it is observed that as the water depths increase, the settlement decreases. This suggests that deeper groundwater levels contribute to reduced settlement and potentially enhance the overall stability of the structure^50^. When the groundwater table is at a depth of 4 m, the settlement is measured to be 50 mm. However, when the groundwater table is deeper at 21 m, the settlement decreases to 45 mm, indicating a change of -10% in settlement. This change suggests that as the groundwater table goes deeper beyond 21 m, a noticeable difference in settlement becomes evident.

#### Variation of a factor of safety and settlement concerning the shape of pile cap

**Figure 12:**
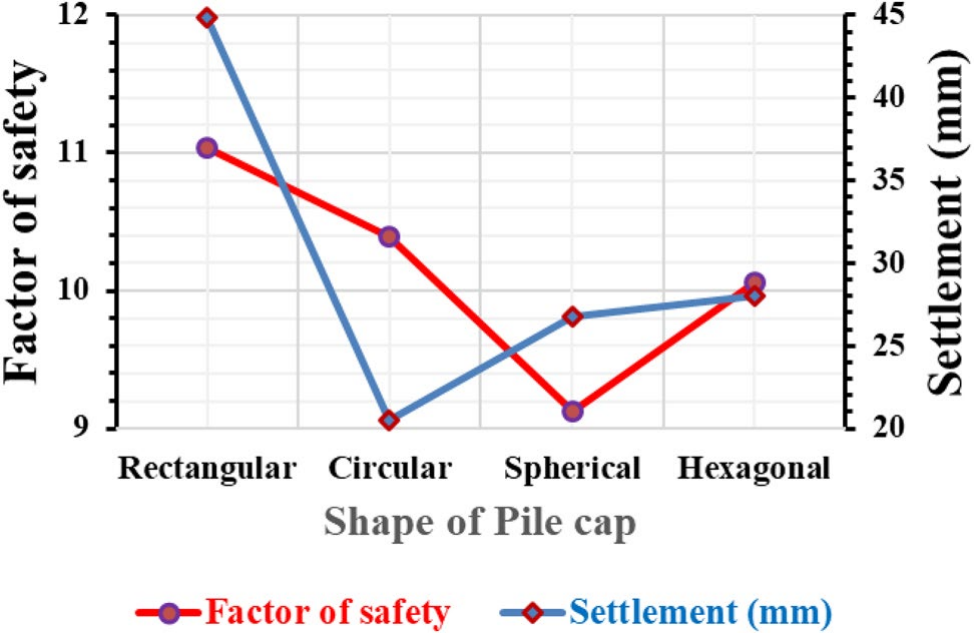
Pile group settlement and factor of safety due to change in the shape of pile cap.

As different types of piles serve different purposes and are utilized in various working conditions, pile caps also vary in shape accordingly. This allows for the efficient design of pile caps that occupy minimal building area while serving their intended function effectively. In our analysis, we considered several shapes for pile caps, including rectangular, circular, spherical, and hexagonal. Figure 12 provides data on their factor of safety and settlement (measured in mm). Upon analysis, it was observed that the circular pile cap exhibited the least settlement and had a higher factor safety compared to the other shapes. On the other hand, the rectangular pile cap experienced a greater settlement than the circular pile cap. Therefore, attaining the desired functionality for rectangular pile caps requires careful analysis and design to ensure optimal performance.

#### Variation of a factor of safety and settlement concerning different soil types

**Figure 13:**
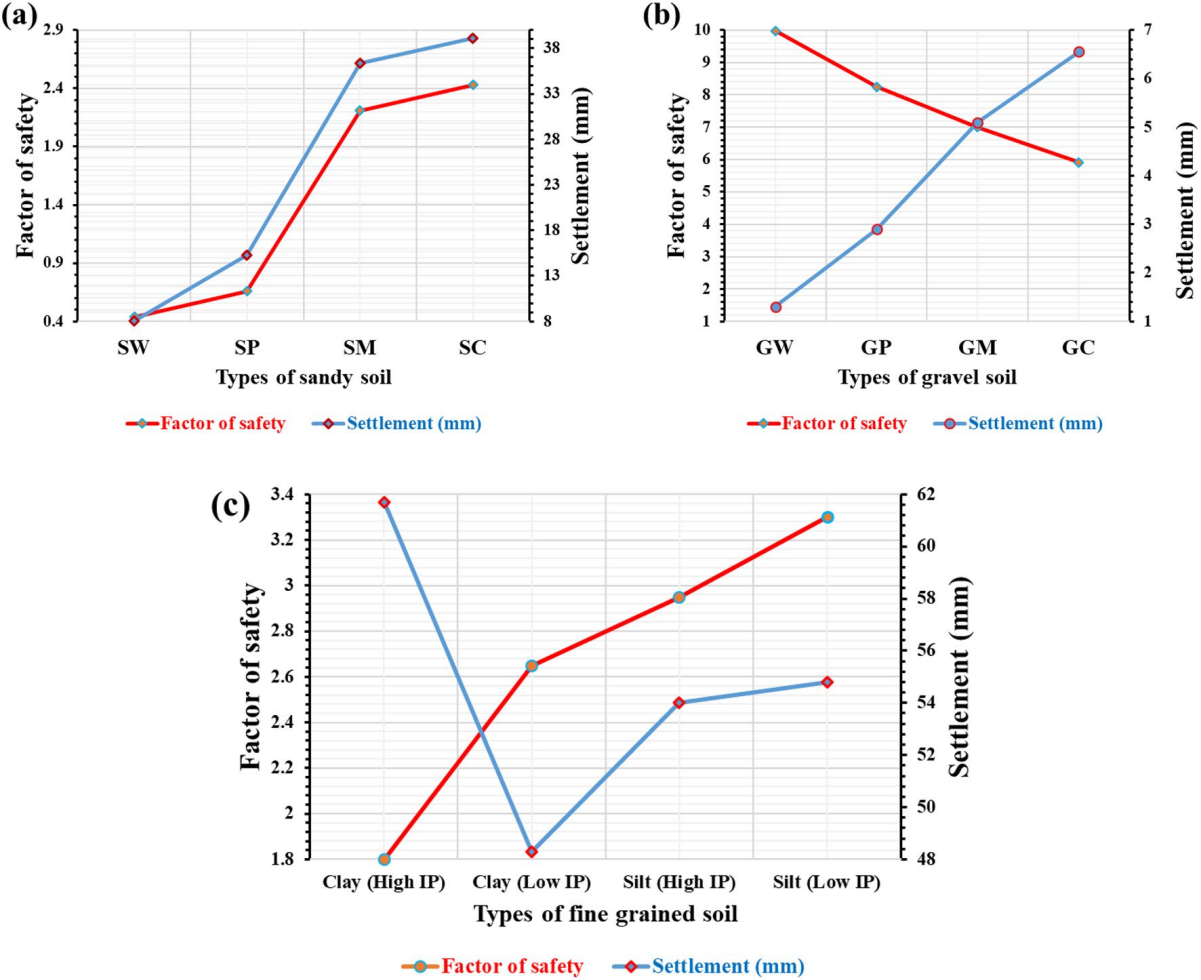
Pile group settlement and factor of safety deviations depending on (**a**) the change in sandy soil type, (**b**) the change in granular soil type, (**c**) the change in fine-grained soil type.

This analysis focused on categorizing soil into three types: sandy, granular, and fine-grained. In the sandy soil category, there are various subtypes including well-graded, poorly-graded, silty, and clayey sand soil. Granular soils are classified into four types: well-graded gravel, poorly-graded gravel, silty gravel, and clayey gravel. Fine-grained soils consist of clay with high plasticity, clay with low plasticity, silt with high plasticity, and silt with low plasticity. After conducting a numerical examination of pile groups, several observations were made. Clay soil demonstrated the highest settlement value compared to sandy and silty soil types. Conversely, silty soil had the lowest settlement value due to its higher degree of compaction and smaller void ratio. Among all soil types, well-graded gravel exhibited the highest factor of safety and the least settling. Figure 13 illustrates the impact of changes in sandy soil, gravel soil, and fine-grained soil granular soil types on the factor of safety and settlements (for the abbreviation of the soil types, refer to Tables 3 and 4).

### Result of study for Piled Raft using PLAXIS 2D

#### Variation of deformation concerning surcharge load on Sand

**Figure 14:**
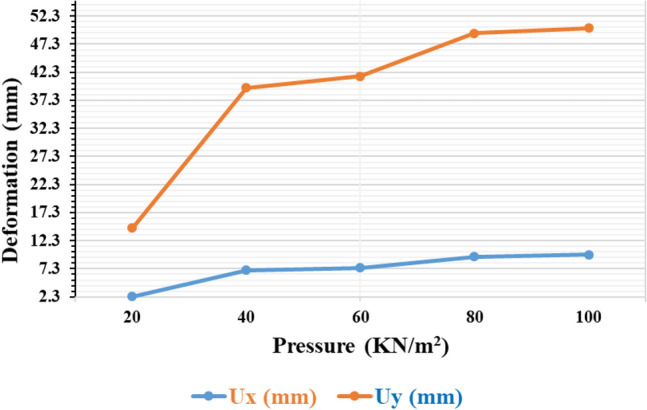
Piled raft deformation with a varying load on sandy soil.

The deformation is measured in terms of horizontal deformation (Ux) and vertical deformation (Uy). Figure 14 illustrates the deformation of piled rafts on sandy soil under varying load conditions. It is evident from the figure that both Ux exhibit significant variations across different surcharge values, specifically at 20 kN/m^2^, 40 kN/m^2^, 60 kN/m^2^, 80 kN/m^2^, and 100 kN/m^2^. Upon closer observation, it becomes apparent that as the applied load increases, the vertical deformation (Uy) demonstrates a greater degree of variability compared to the horizontal deformation (Ux). This distinction can be attributed to the fact that sandy soil possesses a relatively higher side or frictional resistance in comparison to base resistance due to its low cohesiveness. As a result, the soil is more effective at resisting vertical deformation, leading to the increased variance observed in Uy. In the initial increase in the pressure, there is a notable deformation observed along the horizontal and vertical axis.

#### Variation of deformation concerning surcharge load on clay soil

**Figure 15:**
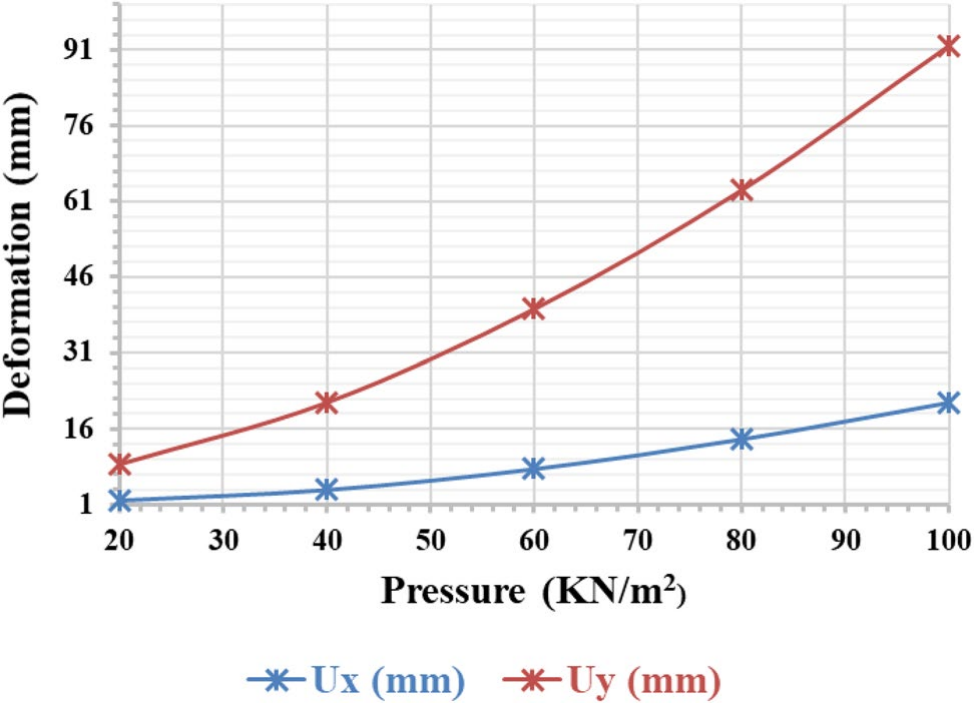
Piled raft deformational changes in varying clay soil.

Figure 15 illustrates the deformation of piled rafts on clay soil under varying load conditions. The horizontal deformation Ux and vertical deformation Uy in pile raft when the surcharge is 20 kN/m^2^, 40 kN/m^2^, 60 kN/m^2^, 80 kN/m^2^, and 100 kN/m^2^, are analyzed on clay soil, the deformation shows almost uniform changes for the applied loads. This means that as the load on the foundation system fluctuates within the same gap difference, the earth beneath the raft may deform, resulting in even settling and significant structural damage. Clay soils pose a particular issue due to their limited bearing capacity and high compressibility. Accordingly, it is reasonable to assume that as the surcharge load increases, the soil’s lateral displacement will cause an increase in horizontal deformation (Ux). Additionally, when the soil contracts under the load, the vertical deformation (Uy) will increase. These deformations should be considered when designing a foundation on clay soils to ensure the stability and integrity of the structure.

#### Variation of deformation on sandy soil with different densities

**Figure 16:**
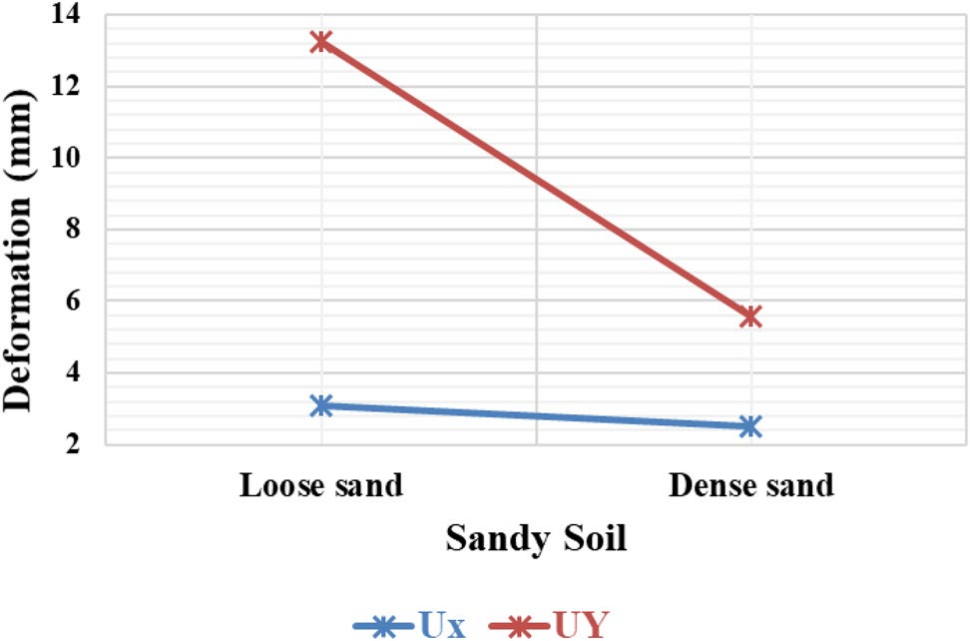
Horizontal and vertical deformation of the piled raft in sandy soil.

The results from numerical analysis demonstrated some interesting findings. In Fig. 16, the investigation focused on analyzing the impact of loose and dense sandy soil on the deformation of piled rafts. Firstly, it was observed that dense sand exhibited comparatively lower deformation, both horizontally and vertically, indicating higher resistance compared to loose sandy soil. Moreover, the analysis also highlighted that vertical deformation resulted in a greater variation in values compared to horizontal deformation. This implies that the extent of vertical deformation can vary significantly across different areas, whereas horizontal deformation shows more consistent behaviour. These findings suggest that the density of sandy soil plays a crucial role in determining the overall deformation characteristics of piled rafts. Dense sand, due to its denser nature, demonstrates enhanced resistance against deformation^51^. Additionally, the variation in vertical deformation values underscores the need for careful consideration and assessment of the specific conditions and properties of sandy soil when designing and constructing piled rafts.

#### Variation of deformation on clayey soil with different stiffness

**Figure 17:**
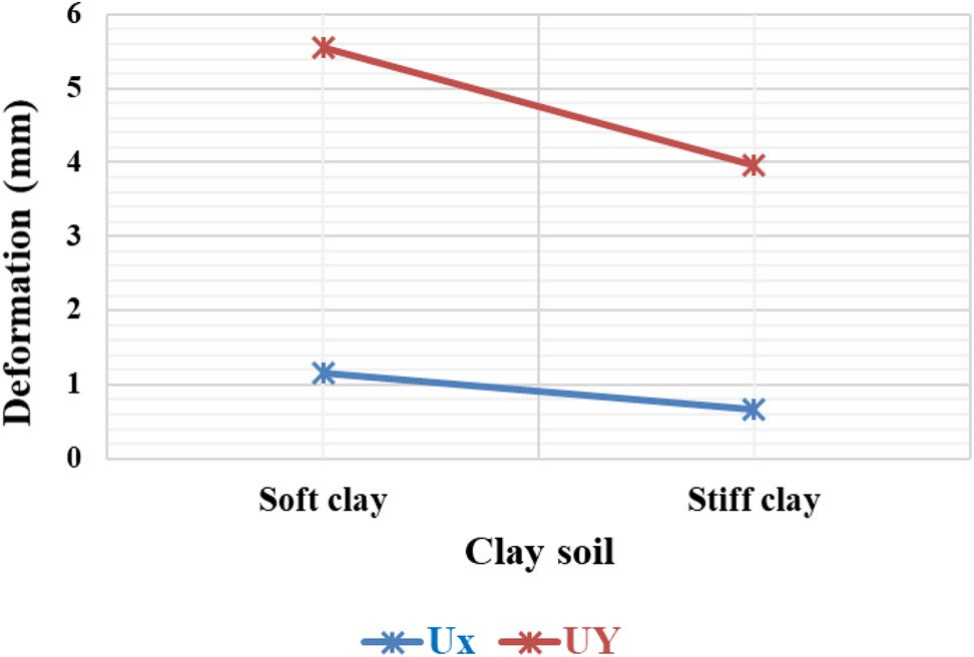
Horizontal and vertical deformation of the piled raft in clay soils.

Figure 17 illustrates the deformation of the piled raft caused by variations in the stiffness of the clay. It shows that the horizontal and vertical deformation of the stiff clay soil is comparatively lower than that of the soft clay soil. This suggests that stiff clay soil possesses a greater capacity to bear loads compared to soft clay soil.

### Result of study for footing using PLAXIS 2D

#### Variation of deformation concerning surcharge

**Figure 18:**
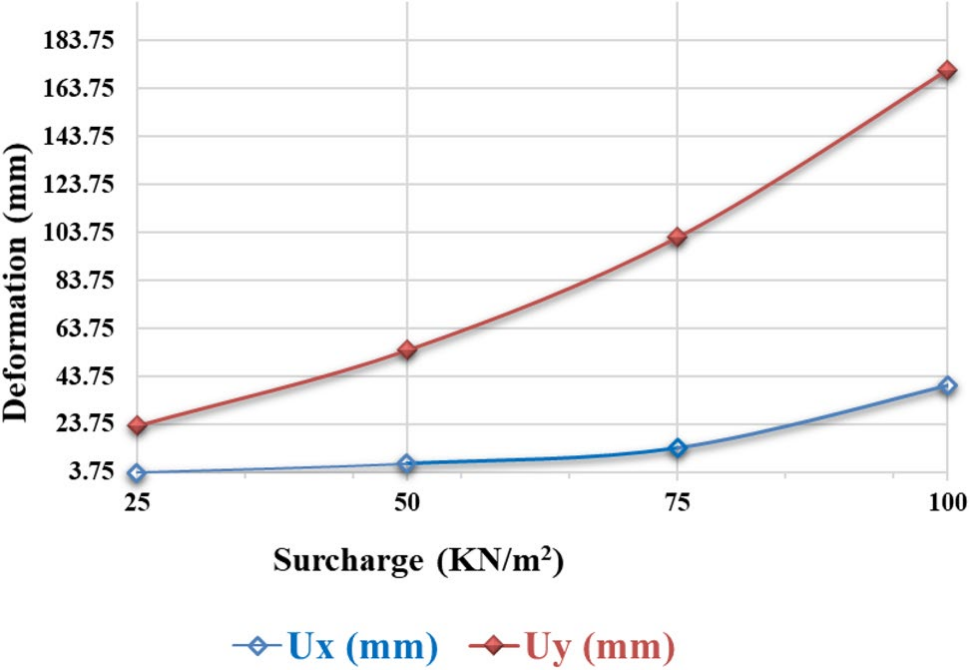
Horizontal and vertical deformation of footing with a variation in surcharge.

Figure 18 represents the varying surcharge develops, both horizontal and vertical deformations increase, however, the vertical deformation increases at a faster pace than the horizontal. In general, the magnitude of deformation, both horizontally and vertically, increases with the applied load on the footing. However, various factors can affect the rate of deformation. Vertical deformation often surpasses horizontal deformation due to the inherent weakness of soils in the vertical direction. Under the same applied surcharge load, vertical deformation is approximately 84% greater than horizontal deformation. This disparity arises from the horizontal layers in which soils are typically deposited, providing them with additional cohesion and strength. When a load is applied, the soil directly beneath the footing experiences vertical compression, leading to faster deformation compared to the surrounding soil in the horizontal direction. Furthermore, soil consolidation and compressibility play significant roles in vertical deformation, causing the soil to settle more rapidly.

#### Variation of deformation in sandy soil with different densities

**Figure 19:**
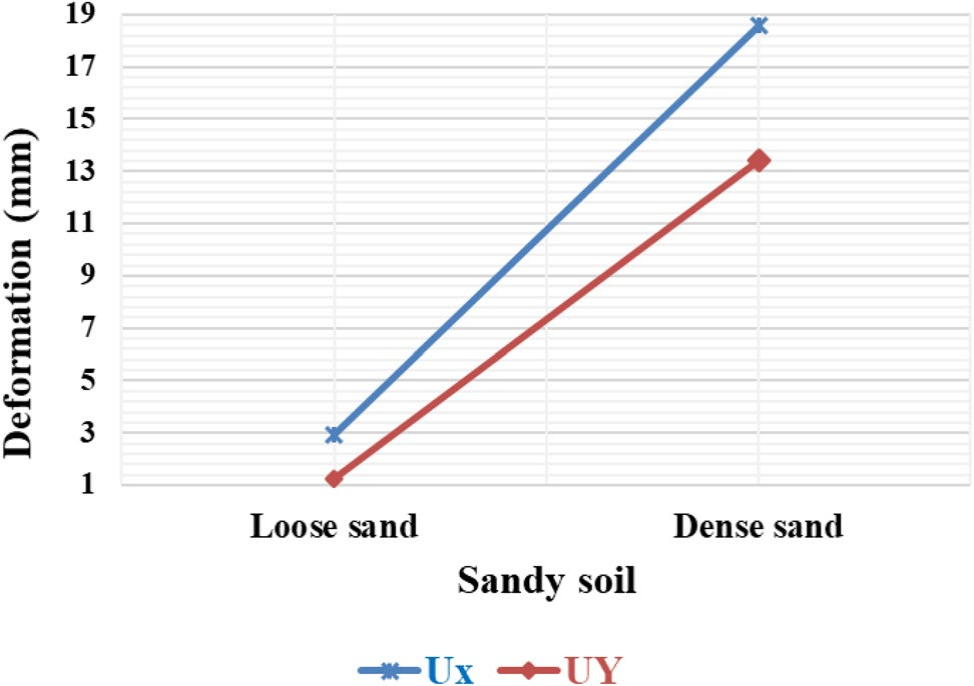
Horizontal and vertical deformation of footing in Sandy soils.

Based on the findings showcased in Fig. 19, it can be observed that dense sand exhibits higher resistance compared to loose sand. The investigation specifically highlights that when compared to loose sandy soil, dense sand experiences minimal horizontal and vertical deformation. It is important to note that in this study, the footing was placed in sandy soil, resulting in greater vertical displacement as opposed to horizontal deformation.

#### Variation of deformation in clay soil concerning different stiffness

**Figure 20:**
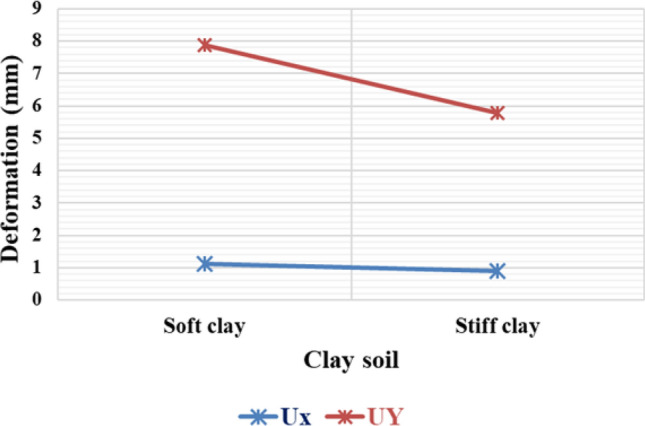
Horizontal and vertical deformation of footing in clayey soils.

Due to the cohesive nature of clay soil, it primarily exhibits resistance through cohesion rather than friction. As a result, horizontal deformations in clayey soil tend to be relatively linear, regardless of whether the clay is soft or hard. In terms of vertical deformations, stiff clay soil experiences less displacement compared to soft clay soil. This suggests that stiff clay soil generally has a higher load-bearing capacity than soft clay soil. The relationship between clay stiffness and footing deformation can be observed in Fig. 20.

### FEM results validation

**Figure 21:**
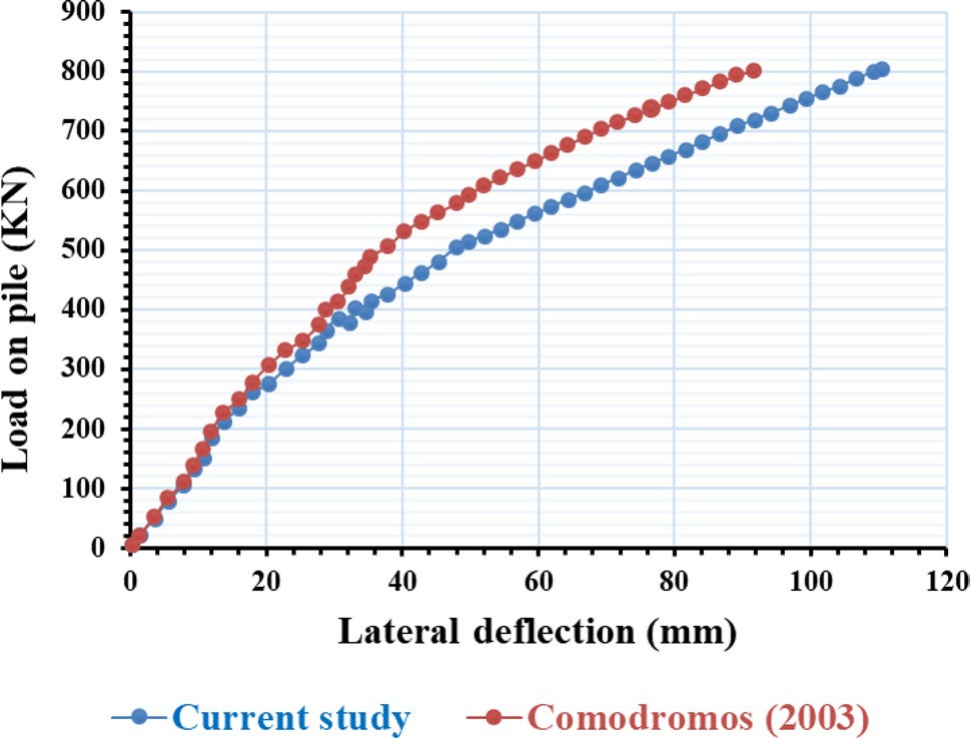
Comparison of published experimental results with the present study.

To validate the entire methodology, GEO5 conducted a simulation similar to the experimental assessment carried out by Comodromos^46^. In their study, Comodromos^46^ examined the reactivity of a drilled pile measuring 52 m in length and 1 m in diameter, which was implemented at a bridge location in Greece for a case study. The analysis focused solely on lateral loads. The subsurface conditions consisted of 36 m of soft clay, followed by 12 m of hard clay, with a layer of thick sand gravel on top. The geotechnical properties of the soil examined by Comodromos^46^ are summarized in Table 6. In the present investigation, the same study was replicated and analyzed using GEO5 to validate the analysis capabilities of the software. The soil parameters for the various layers and piles employed in this investigation matched those described by Comodromos^46^. The study compares the experimental results with the numerical estimates using the same sequence of load application as depicted in Fig. 21. In comparison to the experimental data, the numerical estimates in this study consistently underestimate the lateral capacity of the pile at all deflection levels. The maximum deviation of 10% was observed at a lateral displacement of 100 mm. Consequently, the 3D numerical findings align with the experimental observations of Comodromos^46^.

**Table 6:** Geotechnical parameters of soil strata as determined by Comodroms (2003)^46^.

Soil Layer	Depth (m)	Unit weight c (kN/m^3^)	Undrained Shear strength C_u_ (kPa)	Friction angle, Φ (degrees)	Shear modulus, G (MPa)
Soft silt clay	0–36	20	27	0	243
Medium stiff clay	36–48	20	110	0	335
Very dense sandy gravel	48–70	20	0	40	24

## Conclusions

In conclusion, the paper has provided a comprehensive analysis of numerical concepts for footing, pile group, and pile raft using GEO5 and PLAXIS 2D FEM-based software.

- The study has revealed that the load parameter in piled rafts has a significant impact on deformation and surpasses horizontal deformation in sandy, fine-grained, and granular types of soil. Within clay soil, soft clay exhibits more deformation compared to stiff clay, while loose sand experiences more deformation than dense sand, highlighting a difference in their carrying capacities.
- The investigation of footing analysis with varying pressure has indicated that vertical deformation exceeds horizontal deformation under the same pressure conditions.
- In the pile group analysis, the study has found that increasing pile length positively affects the ultimate capacity of the foundation. Additionally, the study discovered that minimizing settling by more than 10% by increasing pile length and thickness by 50% had a favourable impact on the ultimate capacity of the foundation.
- Circular pile shapes have shown less settling compared to rectangular shapes whereas rectangular pile cap showed greater settlement than the circular pile cap.
- Conversely, when considering diverse soil types, clay with a high plastic index exhibits significant settling among fine-grained soil. Graded granular soil, on the other hand, demonstrates the highest factor of safety and the least settlement.
- The safety factor rose together with the length of the piles. Accordingly, longer piles provide a greater degree of stability and support. It is crucial to take pile length into account when building pile groups in silty gravel soil because longer piles can greatly increase stability and decrease settlement.
- It has been found that settlement reduces as water depth rises. This shows that higher groundwater levels contribute to less settling and may even increase the structure's overall stability.
- The vertical deformation Uy was found to show a greater degree of fluctuation as the applied load increases when compared to that of horizontal deformation (Ux).
- It was concluded that an increase in the horizontal deformation (Ux) will result from the soil's lateral displacement as the surcharge load increases. As the soil will compress beneath the load, increasing the vertical deformation (Uy).
- In comparison to loose sandy soil, dense sand showed relatively less deformation, both horizontally and vertically, indicating stronger resilience. Additionally, the investigation showed that compared to horizontal deformation, vertical deformation caused a higher fluctuation in values.
- In comparison to soft clay soil, stiff clay soil exhibits less horizontal and vertical deformation. This shows that compared to soft clay soil, stiff clay soil has a higher capacity to support loads.
- Vertical displacement was 84% larger than horizontal deformation under the same applied surcharge load. This discrepancy resulted from the horizontal strata that are commonly used to deposit soils, giving them more cohesiveness and strength.
- However, this study is restricted to numerical analysis with no experimental investigation for the study; hence future prospective that would be explored detailing physical modelling and experimental study, can be conducted a case studies to unfold those more comphrend conclusion.
- These findings have important implications for achieving cost-effective design in piled raft foundations and should encourage further research in this area. Overall, this study provides valuable guidance for engineers and researchers seeking optimal designs for piled raft foundations, leading to more efficient and reliable construction practices.

In the current study, we have primarily focused on the analysis and design of a piled-raft foundation system. Moreover, an interesting avenue for future research lies in investigating the intricate mechanics of load sharing between the raft and the individual piles within the system. Also, evaluation of the FOS in the context of piled-raft foundation systems considering different parameters such as pile spacing, pile diameter, raft thickness, and structural loading. The results of such an investigation would provide valuable design guidelines, enabling engineers to make informed decisions regarding the selection and optimization of parameters to achieve desired levels of safety and performance in piled-raft foundations.

## Data Availability

Data will be made available on request from the corresponding author.

## References

[1] Ateş B, Şadoğlu E (2022). Experimental and numerical investigation of load sharing ratio for piled raft foundation in granular soils. KSCE J. Civ. Eng..

[2] Ateş B, Şadoglu E (2022). Experimental and numerical investigation for vertical stress increments of model piled raft foundation in sandy soil. Iran. J. Sci. Technol. Trans. Civ. Eng..

[3] Deb P, Pal SK (2021). Interaction behavior and load sharing pattern of piled raft using nonlinear regression and LM algorithm-based artificial neural network. Front. Struct. Civ. Eng..

[4] Deb P, Debnath B, Reang RB, Pal SK (2022). Structural analysis of piled raft foundation in soft soil: An experimental simulation and parametric study with numerical method. Ocean Eng..

[5] Wulandari PS, Tjandra D (2015). Analysis of geotextile reinforced road embankment using PLAXIS 2D. Proc. Eng..

[6] De Moel M, Bach PM, Bouazza A, Singh RM, Sun JO (2010). Technological advances and applications of geothermal energy pile foundations and their feasibility in Australia. Renew. Sustain. Energy Rev..

[7] Sánchez-Garrido AJ, Navarro IJ, Yepes V (2022). Evaluating the sustainability of soil improvement techniques in foundation substructures. J. Clean. Prod..

[8] Hamed M, Emirler B, Canakci H, Yildiz A (2020). 3D numerical modeling of a single pipe pile under axial compression embedded in organic soil. Geotech. Geol. Eng..

[9] Li Y, Wang J, Wang Q, Yang K (2011). Optimization design study on variable stiffness of the pile raft foundation in high-rise building. Adv. Mater. Res..

[10] Long H (2021). Nonlinear study on the structure-soil-structure interaction of seismic response among high-rise buildings. Eng. Struct..

[11] Rabiei M, Choobbasti AJ (2016). Piled raft design strategies for high rise buildings. Geotech. Geol. Eng..

[12] Hemada AA, National B (2017). Numerical modelling of vertically loaded piled raft foundation by Faculty of Engineering at Cairo University in partial fulfillment of the requirements for the degree of. Rafting.

[13] Poulos HG (2015). The design of foundations for high-rise buildings. ICE Proc. Civ. Eng..

[14] Poulos HG (2016). Tall building foundations: design methods and applications. Innov. Infrastruct. Solut..

[15] Ors DM, Ebid AM, Mahdi HA (2022). Evaluating the lateral subgrade reaction of soil using horizontal pile load test results. Ain Shams Eng. J..

[16] Chen HY, Feng ZJ, Li T, Bai SF, Zhang C (2021). Study on the vertical bearing performance of pile across cave and sensitivity of three parameters. Sci. Rep..

[17] He L (2022). A case study on the bearing characteristics of a bottom uplift pile in a layered foundation. Sci. Rep..

[18] Xing H, Liu L (2018). Field tests on influencing factors of negative skin friction for pile foundations in collapsible Loess regions. Int. J. Civ. Eng..

[19] Cui K, Feng J, Zhu C (2018). A study on the mechanisms of interaction between deep foundation pits and the pile foundations of adjacent skewed arches as well as methods for deformation control. Complexity.

[20] Poulos HG, Small JC, Chow H (2011). Piled raft foundations for tall buildings. Geotech. Eng..

[21] Modak R, Singh B (2022). A parametric study of large piled raft foundations on clay soil. Ocean Eng..

[22] Alnuaim AM, El Naggar H, El Naggar MH (2017). Evaluation of piled raft performance using a verified 3D nonlinear numerical model. Geotech. Geol. Eng..

[23] Sinha A, Hanna AM (2017). 3D numerical model for piled raft foundation. Int. J. Geomech..

[24] Elwakil, A. Z., & Azzam, W. R. (2016). Experimental and numerical study of piled raft system. Alexandria Engineering Journal, 55(1), 547–560.

[25] Ates B, Şadoğlu E (2021). Experimental investigation of optimum piles spacing for piled raft foundation in sandy soils. Tek. Dergi.

[26] Chore H, Ingle R, Sawant VA (2014). Non linear soil structure interaction of space frame-pile foundation-soil system. Struct. Eng. Mech..

[27] Keskin MS, Laman M (2013). Model studies of bearing capacity of strip footing on sand slope. KSCE J. Civ. Eng..

[28] Bhaduri A, Choudhury D (2023). Displacement-based finite element approach on analysing flexible combined pile–raft foundation in layered soil. Can. Geotech. J..

[29] Hazelton P, Murphy B (2016). Interpreting Soil Test Results: What Do All the Numbers Mean?.

[30] Kim D, Jeong S, Park J (2020). Analysis on shaft resistance of the steel pipe prebored and precast piles based on field load-transfer curves and finite element method. Soils Found..

[31] Erdogan, F. *Fracture Mechanics*. Vol. 25. 1220 (ASTM International, 1995).

[32] Emirler B, Tolun M, Yildiz A (2020). Investigation on determining uplift capacity and failure mechanism of the pile groups in sand. Ocean Eng..

[33] Yosef TY, Fang C, Faller RK, Kim S (2023). A multi-material ALE model for investigating impact dynamics of pile-soil systems. Soil Dyn. Earthq. Eng..

[34] Meyerhof GG (1976). Bearing capacity and settlement of pile foundations. ASCE J. Geotech. Eng. Div..

[35] Bowles, J.E. *Foundation Analysis and Design*. 478999 (1997).

[36] Bentley SP (2016). Soil Properies and Their correlations.

[37] Abbas Al-Shamary JM, Chik Z, Taha MR (2018). Modeling the lateral response of pile groups in cohesionless and cohesive soils. Int. J. Geo-Eng..

[38] Liu B, Wang X, Liu C, Kong J (2023). Effect of relative stiffness of pile and soil on pile group effect. J. Mar. Sci. Eng..

[39] Haskell J, Madabhushi G, Cubrinovski M (2011). Effect of pile spacing on the behaviour of a pile group in laterally spreading soil. Int. Conf. Earthq. Geotech. Eng..

[40] Zhang Y, Zhou LP, Wang MY, Ding X, Wang C (2021). Experimental study on the negative skin friction of the pile group induced by rising and lowering the groundwater level. Adv. Civ. Eng..

[41] Yang S, Ren X, Zhang J (2011). Study on embedded length of piles for slope reinforced with one row of piles. J. Rock Mech. Geotech. Eng..

[42] He CL, Gong CZ (2014). Influence of pile cap thickness on the bearing characteristics of group piles foundation. Appl. Mech. Mater..

[43] Tian D, Zheng H (2023). The generalized Mohr–Coulomb failure criterion. Appl. Sci..

[44] Brinkgreve, R.B.J., Broere, W. & Waterman, D. *Plaxis 2D Manual—Version 8* (2006).

[45] Jones K, Sun M, Lin C (2022). Numerical analysis of group effects of a large pile group under lateral loading. Comput. Geotech..

[46] Ahmed, S., Sadique, M. R. & Sawant, V. A. *Response of Pile Foundation to Horizontal Load : A Review Response of Pile Foundation to Horizontal Load : A Review*. (2017).

[47] Duncan JM (2000). Factors of safety and reliability in geotechnical engineering. J. Geotech. Geoenviron. Eng..

[48] Park D, Lee J (2015). Comparative analysis of various interaction effects for piled rafts in sands using centrifuge tests. J. Geotech. Geoenviron. Eng..

[49] El-mossallamy Y, Prof A, Allee B, Darmstadt D (2008). Modelling the behaviour of piled raft applying Plaxis 3D Foundation version 2. Plaxis Bull..

[50] Alsabhan AH, Sadique R, Alam S (2022). Materials today : Proceedings response of deep foundation due to fluctuation in ground water table. Mater. Today Proc..

[51] Tilahun MW, Assefa SM (2023). Analysis of piled raft foundation behaviors in sandy soils using a numerical approach. Model. Earth Syst. Environ..

